# Supplementary material for: Fixational eye movements enhance the precision of visual information transmitted by the primate retina
Source: Nat Commun. 2024 Sep 11;15:7964. doi: 10.1038/s41467-024-52304-7 (PMC11390888; doi:10.1038/s41467-024-52304-7)
Supplement: Supplementary file 3 — Reporting Summary [file 41467_2024_52304_MOESM3_ESM.pdf]

nature portfolio

Corresponding author(s): Wu, Chichilnisky

Last updated by author(s): Jul 17, 2024

Reporting Summary

Nature Portfolio wishes to improve the reproducibility of the work that we publish. This form provides structure for consistency and transparency in reporting. For further information on Nature Portfolio policies, see our Editorial Policies and the Editorial Policy Checklist.

Statistics

For all statistical analyses, confirm that the following items are present in the figure legend, table legend, main text, or Methods section.

n/a	Confirmed	
☐	☒	The exact sample size (*n*) for each experimental group/condition, given as a discrete number and unit of measurement
☐	☐	A statement on whether measurements were taken from distinct samples or whether the same sample was measured repeatedly
☐	☒	The statistical test(s) used AND whether they are one- or two-sided<br>*Only common tests should be described solely by name; describe more complex techniques in the Methods section.*
☐	☒	A description of all covariates tested
☒	☐	A description of any assumptions or corrections, such as tests of normality and adjustment for multiple comparisons
☐	☒	A full description of the statistical parameters including central tendency (e.g. means) or other basic estimates (e.g. regression coefficient) AND variation (e.g. standard deviation) or associated estimates of uncertainty (e.g. confidence intervals)
☐	☒	For null hypothesis testing, the test statistic (e.g. *F*, *t*, *r*) with confidence intervals, effect sizes, degrees of freedom and *P* value noted<br>*Give P values as exact values whenever suitable.*
☐	☒	For Bayesian analysis, information on the choice of priors and Markov chain Monte Carlo settings
☒	☐	For hierarchical and complex designs, identification of the appropriate level for tests and full reporting of outcomes
☐	☒	Estimates of effect sizes (e.g. Cohen's *d*, Pearson's *r*), indicating how they were calculated

Our web collection on statistics for biologists contains articles on many of the points above.

Software and code

Policy information about availability of computer code

Data collection	Custom software for the multi-electrode array was used to help perform the neural recordings. Custom software was used to present the visual stimulus to the retina on a computer display.
Data analysis	YASS spike sorter (https://github.com/paninski-lab/yass, version 2.0)<br>Custom proprietary software for loading spike-sorted data<br>Custom software for fitting encoding models and for performing reconstructions (https://github.com/wueric/wu-nature-comms-2024)<br>Custom software used numpy version 1.25.2, pytorch version 2.0.1, scipy 1.11.1, matplotlib 3.7.1, CUDA 12.2<br>statsmodels 0.14.0, shapely 2.0.1, scikit-image 0.20.0, tqdm 4.65.0, h5py 3.9.0

For manuscripts utilizing custom algorithms or software that are central to the research but not yet described in published literature, software must be made available to editors and reviewers. We strongly encourage code deposition in a community repository (e.g. GitHub). See the Nature Portfolio guidelines for submitting code & software for further information.

Data

Policy information about availability of data

All manuscripts must include a data availability statement. This statement should provide the following information, where applicable:
- Accession codes, unique identifiers, or web links for publicly available datasets
- A description of any restrictions on data availability
- For clinical datasets or third party data, please ensure that the statement adheres to our policy

Source data are provided with this paper. A limited dataset containing spike-sorted spike trains and visual stimuli sufficient for generating the example reconstruction images in the figures has been deposited in a Figshare repository (https://doi.org/10.6084/m9.figshare.23929941). The raw voltage traces are not available due to their large size (> 5 Tb), the complexity of the data processing pipeline, and ongoing use of the data for unpublished research.

Research involving human participants, their data, or biological material

Policy information about studies with human participants or human data. See also policy information about sex, gender (identity/presentation), and sexual orientation and race, ethnicity and racism.

Reporting on sex and gender	No human participants, data, or biological material.
Reporting on race, ethnicity, or other socially relevant groupings	Not relevant.
Population characteristics	Not relevant.
Recruitment	Not relevant.
Ethics oversight	Not relevant. No human participants, data, or biological material.

Note that full information on the approval of the study protocol must also be provided in the manuscript.

Field-specific reporting

Please select the one below that is the best fit for your research. If you are not sure, read the appropriate sections before making your selection.

☒ Life sciences ☐ Behavioural & social sciences ☐ Ecological, evolutionary & environmental sciences

For a reference copy of the document with all sections, see nature.com/documents/nr-reporting-summary-flat.pdf

Life sciences study design

All studies must disclose on these points even when the disclosure is negative.

Sample size	Sample sizes were determined by the amount of experimental data that was feasible to collect.
Data exclusions	No data were excluded from the analyses.
Replication	All attempts at replication were successful.
Randomization	No randomization was used, as all available data were used for the analyses.
Blinding	No blinding was relevant to this study.

Reporting for specific materials, systems and methods

We require information from authors about some types of materials, experimental systems and methods used in many studies. Here, indicate whether each material, system or method listed is relevant to your study. If you are not sure if a list item applies to your research, read the appropriate section before selecting a response.

Materials & experimental systems

n/a	Involved in the study
☒	☐ Antibodies
☒	☐ Eukaryotic cell lines
☒	☐ Palaeontology and archaeology
☐	☒ Animals and other organisms
☒	☐ Clinical data
☒	☐ Dual use research of concern
☒	☐ Plants

Methods

n/a	Involved in the study
☒	☐ ChIP-seq
☒	☐ Flow cytometry
☒	☐ MRI-based neuroimaging

Animals and other research organisms

Policy information about studies involving animals; ARRIVE guidelines recommended for reporting animal research, and Sex and Gender in Research

Laboratory animals	Macaque monkeys (Macaca mulatta, Macaca fasicularis, ages 12, 15, 12.5, 15-20, 18 years old, respectively)
Wild animals	None
Reporting on sex	Though experimental animals were not chosen by sex, only male animals were used. No sex-based analyses were performed.
Field-collected samples	No field-collected samples.
Ethics oversight	Animal experimentation: Eyes were removed from terminally anesthetized macaque monkeys (Macaca mulatta, Macaca fascicularis) used by other laboratories in the course of their experiments, in accordance with the Institutional Animal Care and Use Committee guidelines. All of the animals were handled according to approved institutional animal care and use committee (IACUC) protocols (#28860) of the Stanford University. The protocol was approved by the Administrative Panel on Laboratory Animal Care of the Stanford University (Assurance Number: A3213-01).

Note that full information on the approval of the study protocol must also be provided in the manuscript.

Plants

Seed stocks	None
Novel plant genotypes	None
Authentication	None
